# The association of periodontal diseases and Sjogren’s syndrome: A systematic review and meta-analysis

**DOI:** 10.3389/fmed.2022.904638

**Published:** 2023-01-05

**Authors:** Bo Yang, Xuefei Pang, Jiazhong Guan, Xu Liu, Xiting Li, Yan Wang, Zhuofan Chen, Bin Cheng

**Affiliations:** ^1^Hospital of Stomatology, Guanghua School of Stomatology, Sun Yat-sen University, Guangdong Provincial Key Laboratory of Stomatology, Guangzhou, China; ^2^Guangdong Provincial Key Laboratory of Biomedical Imaging and Guangdong Provincial Engineering Research Center of Molecular Imaging, Department of Infectious Disease, The Fifth Affiliated Hospital, Sun Yat-sen University, Zhuhai, China

**Keywords:** periodontal disease, observational study, systematic review, meta-analysis, Sjogren’s syndrome

## Abstract

**Background:**

The relationship between periodontal diseases and Sjogren’s syndrome were found inconsistent in current studies. Our objective is to clarify the relationship between periodontal diseases and Sjogren’s syndrome.

**Methods:**

A systematic review was performed and reported according to Preferred Reporting Items for Systematic Reviews and Meta-Analyses (PRISMA). Electronic databases (EMBASE, PubMed, Web of Science, and Cochrane Library, from inceptions until 24 November 2021) were searched. The Newcastle-Ottawa Scale (NOS) and Agency for Healthcare Research and Quality (AHRQ) were applied to evaluate the quality of studies. Quality assessment of the certainty of evidence was performed based on the Grading of Recommendations Assessment, Development, and Evaluation (GRADE) guidelines. When the output is the ratio, Odds ratio (OR) of periodontal diseases with Sjogren’s syndrome were calculated. When the output is the mean, weighted mean difference (WMD) of periodontal diseases with Sjogren’s syndrome was calculated. We conducted meta-analysis and estimated the pool sensitivity. Begg’s test was used to test the possibility of publication bias. We also carried out meta-regression to clarify the source of heterogeneity (I2 > 50%). Finally, we performed a trial sequential analysis (TSA) to identify the false positive or false negative outcomes that might occur during repeated updates.

**Results:**

21 studies were included in this systematic review, with a total of 11435 subjects. Meta-analysis of 5 studies showed that there is a positive correlation between periodontitis and Sjogren’s syndrome (OR = 2.12, 95% CI = 1.43–3.17; 5 studies, 6927 participants; low certainty of evidence). Meta-analysis of 16 studies showed that the periodontal condition of patients with Sjogren’s syndrome was worse compared with the control group, and the scores of clinical periodontal parameters were relatively high.

**Conclusion:**

Sjogren’s syndrome patients seem to be more likely to be diagnosed with periodontal diseases. However, our results should be interpreted with caution considering the high heterogeneity.

**Systematic review registration:**

[https://www.crd.york.ac.uk/prospero/], identifier [CRD42021261322].

## 1. Introduction

Periodontal disease is an inflammatory and infectious disease. In the early stages of periodontal disease, the main symptoms are red, swollen and bleeding gums. As the disease progresses, the teeth become loose, mainly due to the development of periodontal pockets and absorption of alveolar bone (1). Gingivitis and periodontitis, the most common forms of periodontal diseases, are triggered by a pathogenic microbiota in the subgingival biofilm. They comprise a variety of inflammatory conditions and periodontitis that could lead to tooth loss and contribute to systemic inflammation (2). Periodontitis is very common in adults and it is more serious in the elderly. It is estimated that about 10−15% of the elderly will develop severe periodontitis (3). The microbial immune subversion, the disturbance of the immune microenvironment and the subsequent systemic inflammatory response in periodontal diseases have been examined for decades (4), which may serve as the causative factor of autoimmune diseases. Some studies have reported that adverse periodontal status may aggravate certain autoimmune diseases, such as rheumatoid arthritis (5, 6), systemic lupus erythematosus (7, 8), Sjogren’s syndrome (SS) (9), thrombocytopenia purpura (10). Thus, there is increasing interest in the potential link between periodontal diseases and certain autoimmune diseases.

Sjogren’s syndrome (SS) is an autoimmune disorder with secretory gland dysfunction charactered by dryness of the main mucosal surfaces including the mouth, eyes, nose, and vagina (11, 12). It can occur alone as primary SS (pSS) or be associated with other systemic diseases as secondary SS (sSS) (13). Incidence and prevalence rates of pSS vary widely around the world. The prevalence rate of pSS was 43.03 cases per 100,000 inhabitants across a series of population-based studies in which the overall age of patients was 56.16 years (14). It is worth mentioning that increasing incidences of oligoptyalism in patients with Sjogren’s syndrome have been reported, which affects the removal of dental plaque and ultimately may lead to periodontal diseases.

According to the published literature in the early years, no significant difference could be detected concerning the periodontal status of SS patients, compared with that of the patients with other immune diseases as well as with that of systemically healthy subjects (15–17). However, in recent years, putative links between periodontal diseases and SS have been reported in several studies. For example, Chuang et al. (18) found that the prevalence (74.6% vs. 63.0%, *P* = 0.001) and frequency (median 5.37 vs. 1.45 per year, *P* < 0.001) of dental visits were found higher in patients with pSS and the risk of gingivitis (aIRR 1.43, *P* < 0.001) and periodontitis (aIRR 1.44, *P* < 0.001) was also significantly higher them (18). Results of the above researches contradict previous systematic reviews (19). To systematically ascertain whether patients with SS are more likely to be diagnosed with periodontal disease, we included articles that explored the incidence of periodontal diseases in patients diagnosed with SS compared with subjects without SS and conducted this systematic review.

## 2. Methods

### 2.1. Protocol and registration

This systematic review was conducted according to the Meta-analysis of Observational Studies in Epidemiology guidelines (20), the Preferred Reporting Items for Systematic Reviews and Meta-Analyses standard (PRISMA) (21). It was registered in PROSPERO (CRD42021261322). The research question of this meta-analysis and systematic review was that: is there an association between periodontal disease and SS subjects meeting the following eligibility criteria?

### 2.2. Eligibility criteria

The strategy for search process was conducted using the PEOS model:

(1) P (patient/participants): subjects aged ≥ 14 years and without other related diseases (such as head and neck tumors);
(2) E (exposure): Sjogren’s syndrome [with the criteria such as the American-European Consensus Group Criteria (AECG), the European Community criteria, etc.];
(3) O (outcome): periodontal disease percentage or clinical periodontal parameters;
(4) S (study design): observational study (cohort, case–control, and cross-sectional studies).

### 2.3. Information sources and search strategy

We conducted a systematic literature search in the PubMed, EMBASE, Web of Science, and Cochrane Library (from their inceptions until 24 November 2021) to investigate the relationship between SS and periodontal diseases. To identify all related datasets and the relevant articles, we used search strategies shown in Supplementary Table 1.

### 2.4. Study selection and data collection

All retrieved studies were independently assessed by two researchers (XP and JG) based on inclusion/exclusion criteria. For studies whose titles and abstracts might meet the inclusion criteria, the full text was selected for further evaluation. A third author BY was involved when there was a disagreement between the two researchers (XP and JG).

After identifying the included studies that met the criteria, XP and JG extracted the following information: study characteristics (author/s, year of publication, study design, country, and characteristics of participants); periodontal diseases assessment [by clinical diagnosis, oral examination including bleeding on probing (BOP), plaque index (PI), probing pocket depth (PPD), gingival index (GI), and clinical attachment level (CAL) or self-report]; and SS assessment (by clinical diagnosis or clinical examination).

### 2.5. Risk of bias and applicability assessment

The Newcastle-Ottawa Scale (NOS) was applied for assessing case-control studies and cohort studies. The evaluation criteria were Selection, Comparability and Exposure. Stars were awarded each study (up to 9 stars) for quick visual assessment, Studies awarded with 6 or more stars were defined as high quality research (22, 23). For cross-sectional studies, the Agency for Healthcare Research and Quality (AHRQ) methodology checklist was applied. This is a methodological quality assessment tool using an 11-item checklist, and the AHRQ recommends it for assessment of cross-sectional studies. Article quality was assessed as follows: low quality = 0–3; moderate quality = 4–7; high quality = 8–11.

The results of NOS and AHRQ scores can be used for reference in the quality of evidence grading. Two reviewers (XP and JG) independently evaluated the quality of the articles, and a third author (BY) evaluated the articles in case of disagreement.

### 2.6. Data synthesis

Forest plots were generated to assess the ORs and corresponding 95% CIs or WMDs and 95% CIs across the studies for meta-analysis. The data reported in some studies were standard error of mean (SEM) and we converted them into SD (SEM = SD/√N) (24). Forest plots were generated to intuitively assess the ORs and corresponding 95% CIs in dichotomous variables or weighted mean difference (WMD) and 95% CIs in continuous variables across the articles. Considering the heterogeneity among the included studies, we used a random-effects model.

We assessed the heterogeneity with Cochran’s *Q* test. If the *p*-value was lower than 0.1, the *I*^2^ statistic was used to quantify the statistical heterogeneity. The threshold was determined as Cochrane recommended, that is 0% to 50%: may not be important; 50% to 100%: may represent substantial heterogeneity (25). In addition, we also carried out meta-regression to clarify the source of heterogeneity. Sensitivity analyses were performed by comparing the pooled sensitivity and specificity results when including and excluding studies with high risk of bias. The Begg’s test was used to test the possibility of publication bias. Finally, we performed a trial sequential analysis (TSA) to identify the false positive or false negative outcomes that might occur during repeated updates. The Stata statistical software version 14 and TSA 0.9 was used to analyze the data. *P*-values were two-sided, and the significance level was set at 0.05.

The Grading of Recommendations Assessment, Development, and Evaluation (GRADE) guidelines were used for rating the quality of evidence (26). The degree of certainty of evidence is high, moderate, low or very low. Observational studies are initially rated low by default (26) and are downgraded according to the following pre-specified criteria: risk of bias, inconsistency, inaccuracy, inaccuracy, and publication bias (26).

## 3. Results

### 3.1. Literature search and study characteristics

**FIGURE 1:**
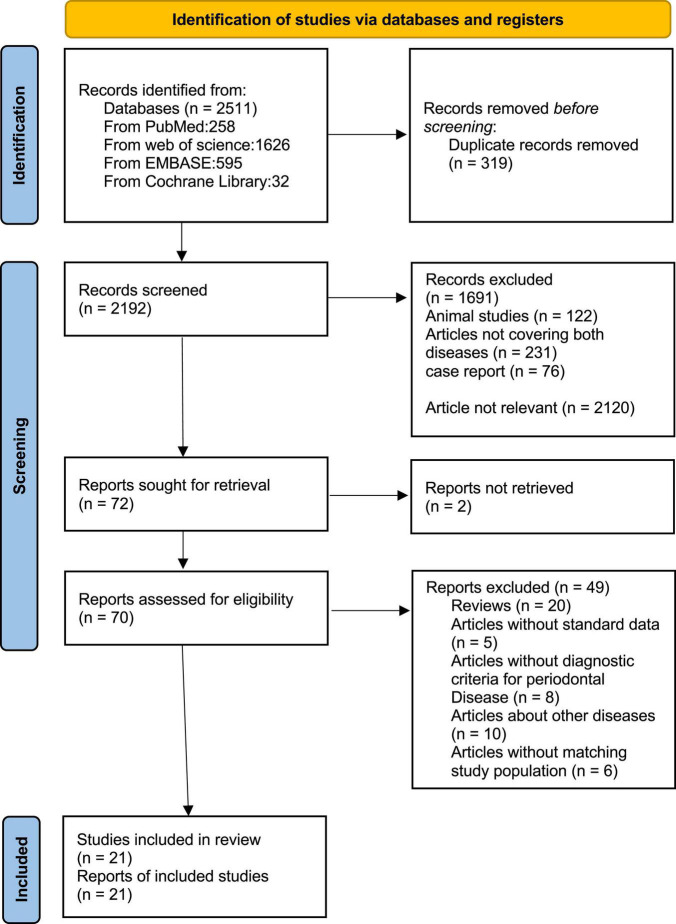
Flow diagram of study selection.

We identified 2511 articles in PubMed, EMBASE, Web of Science, and Cochrane Library. After screening the titles and abstracts of all the articles, 72 articles were selected for further evaluation. Then we reviewed the full text of these articles and excluded 51 articles based on inclusion criteria. In the end, there are 21 articles with 11435 participants meeting the inclusion criteria for the meta-analysis (Figure 1).

Among these 21 articles, 5 articles evaluated the percentage of periodontal diseases in SS subjects and non-SS subjects, and 16 reported the diagnostic criteria scores of clinical periodontal parameters (mean ± SD) in SS subjects and non-SS subjects. Among these latter 16 studies, 7 reported BOP scores, 12 reported PI scores, 12 reported PPD scores, 10 reported GI scores, and 6 reported CAL scores. All included studies were observational, including 3 cohort studies, 4 case-control studies, and 14 cross-sectional studies. The total number of participants was 11,435 (9597 SS patients and 1838 non-SS patients). With regard to the age of the participants, the average age of all studies was over 30 years old. 6 of the studies assessed women separately (16, 27–31), while the rest assessed men and women, and were predominantly female. In the control group, all studies included individuals with no other related diseases. In the diagnostic criteria of SS, International Classification of Diseases, 9th revision, Clinical Modification coding system (ICD-9-CM) (18, 32) was selected as the diagnostic standard in 2 studies, AECG was selected as the diagnostic standard in 8 studies (27, 29, 31, 33–37), and the European classification criteria for SS was selected in 8 studies (16, 17, 28, 38–42). The Copenhagen Criteria was used in 3 studies (31, 41, 43) and other criteria were used in 2 studies (30, 44). Among the diagnostic criteria for periodontal disease, ICD-9-CM was selected in two studies (18, 32), self-report was selected in one study (27), “clinical examination” was only reported in one study without explicit description of examination content (34), and clinical indicators were reported in the rest of the studies (Table 1).

**TABLE 1:** Characteristics of the included studies on the prevalence of periodontal diseases in patients with Sjogren’s syndrome.

References	Study design	Country	Age, years	Periodontal diseases assessment	SS assessment	n (control)	n (SS cases)	Number of participants	Women (%)	Fundings
Chuang et al. (18)	Cohort	China	Range: 20−80	Gingivitis: ICD-9-CM: 523.0, 523.1, and 523.2Periodontitis: ICD-9-CM: 523.3, 523.4, 523.5, and 523.8	ICD-9-CM: 710.2	7090	709	7799	88.9%	−
Albrecht et al. (27)	Cohort	Germany	Patients range: 24−80; mean ± SD: 58.1 ± 12Controls range: 19−76; mean ± SD: 54.1 ± 14	Self-reported	AECG	87	205	292	100%	−
Lu et al. (32)	Cohort	China	Mean ± SD: 54 ± 14	Gingivitis: ICD-9-CM: 523.0–523.9Periodontitis: ICD-9-CM: 523.3–523.5	ICD-9-CM: 710.2	1945	389	2334	90%	−
Ozcaka et al. (33)	Cross-sectional case control	Turkey	Mean ± SD: 51.1 ± 14.1	PPD, PI, BOP	AECG	25	44	69	−	The Research Foundation of Ege University, Izmir, Turkey.
Crincoli et al. (34)	Case–control	Italy	Patients range: 21−82; mean ± SD: 56.06 ± 12.19Controls range: 19−76; mean ± SD: 55.32 ± 12.17	Clinical examination	AECG	72	72	144	97.2%	−
Ergun et al. (44)	Case–control	Turkey	Patients range: 26−78; mean: 53.27Controls range: 25−94; mean: 54.27	PPD, API, BOP	The recently modified internationally agreed-on criteria for SS	37	37	74	−	−
Antoniazzi et al. (17)	Cross-sectional	Brazil	Mean ± SD: 50.1 ± 12.5pSS mean ± SD: 48.1 ± 13.4sSS mean ± SD: 53.8 ± 11.6Controls mean ± SD: 49.8 ± 12.8	PI, GI, CAL, PPD, BOP	The European Community criteria	19	19	38	50%	−
Pedersen et al. (31)	Case–control	Denmark	Patients Mean ± SD: 60 ± 15Controls Mean ± SD: 56 ± 13	PI, GI, PPD	AECG; the Copenhagen criteria	20	20	40	100%	−
Leung et al. (38)	Cross-sectional	China	pSS range: 33−76; mean ± SD: 51.4 ± 14.3sSS range: 27−66; mean ± SD: 43.3 ± 10.9Controls range: 27−75; mean ± SD: 44.0 ± 10.7	PI, CI, CAL	The European Community Diagnostic Criteria	29	51	80	93.33%	CRCG grant from the University of Hong Kong.
Najera et al. (39)	Cross-sectional	United States	Patients range: 28 - 80; mean ± SD: 60.92 ± 13.52Controls range: 30 – 77; mean ± SD: 58.29 ± 12.09	PI, GI, BOP, PPD, CAL	The European Community Criteria	24	25	49	91.84%	−
Tervahartiala et al. (43)	Cross-sectional	Denmark	Patients range: 38−63; mean: 52Controls range: 27−42; mean: 31	PI, PPD, GBI	The Copenhagen criteria	6	8	14	−	The Finnish Academy; the Kordelin Foundation; the Research Foundation for Women; the Finnish Dental Association.
Ambrosio et al. (29)	Cross-sectional	Brazil	Patients Mean ± SD: 52.14 ± 14.1Controls Mean ± SD: 49 ± 6.73	PPD, CAL, BOP, PI	AECG	7	7	14	100%	The SALIVA Research Nucleus of Support (NAP-SALIVA) of the University of São Paulo; the research grants from São Paulo Research Foundation (FAPESP, respectively, 2015/07396-2, and 2013/26381-0); the scholarships from FAPESP (respectively, 2014/06387-7, and 2015/24061-4).
Marton et al. (35)	Cross-sectional	Hungary	Range: 32–76Patients Mean ± SD: 55 ± 11Controls Mean ± SD: 49 ± 15	GBI, PPD, BOP	AECG	43	49	92	92.39%	The Hungarian Scientific Research Fund (OTKA no. T-037776); the Hungarian Medical Research Council (ETT-247/2003).
Kuru et al. (16)	Cross-sectional	England	pSS range: 35−77; mean ± SD: 61.2 ± 14.4sSS range: 43−77; mean ± SD: 60.6 ± 11.8Controls range: 40−77; mean ± SD: 61.8 ± 13.09	PI, GI, BOP, PPD, CAL	The European Community Criteria	11	18	29	100%	−
Pedersen et al. (40)	Cross-sectional	Norway	Patients range: 40−82; Mean: 61.4Controls range: 39−70; Mean: 50	PI, GI, PPD	The European classification criteria for SS	14	16	30	90%	The Ingeborg and Leo Dannin Foundation; the Danish Dental Association Research Foundation (DTF’s Forskningsfond and FUT); the Colgate Research Foundation; the Ib Henriksen Research Foundation.
Pedersen et al. (41)	Cross-sectional	Denmark	Patients range: 40−82; Mean: 64.1Controls range: 39−70; Mean: 64.8	PI, GI, PPD	European classification criteria for SS and the Copenhagen criteria	20	20	40	82.5%	The Zendium Household and Body Care Research Foundation; the Danish Dental Association Research Foundation (DTF’s forskningsfond and FUT).
Tseng (30)	Cross-sectional	United States	Patients Mean: 52.9Controls Mean: 53.7	GI, PI, BI, PPD, CAL	NS	14	14	28	100%	−
Zoppo et al. (42)	Cross-sectional	Venezuela	Patients range: 43−68; Mean ± SD: 54.8 ± 10Controls range: 21−43; Mean ± SD: 32 ± 8.34	GI, PI, PPD	The European classification criteria for SS	6	7	13	69.23%	−
Seck-Diallo et al. (36)	Cross-sectional	Senegal	pSS mean ± SD: 46.7 ± 2.5sSS mean ± SD: 48.2 ± 1.4	PI, GI, PPD, CAL	AECG	103	103	206	91.26%	−
Pers et al. (37)	Cross-sectional	France	Patients range: 44−81Controls range: 39−82	PI, GI, BOP, PPD	AECG	15	15	30	83.3%	−
Rhodus and Michalowicz (28)	Cross-sectional study	United States	Patients range: 43−74; Mean: 56.7Controls range: 32−65; Mean: 52.6	PI, GI, PPD, CAL	The comprehensive European Community Criteria	10	10	20	100%	−

ICD-9-CM, the International Classification of Diseases, 9th revision, Clinical Modification coding system; AECG, the American-European Consensus Group Criteria; BOP, bleeding on probing; PI, plaque index; PPD, probing pocket depth; GI, gingival index; CAL, clinical attachment level; MPD, mean probing depth; CPD, cumulative probing depth; API, approximal plaque index; CI, calculus indices; MAL, mean attachment loss; PAL, probing attachment level; GBI, gingival bleeding index.

The NOS scores and the AHRQ methodology checklist for quality assessment of these studies are shown in Tables 2, 3. The GRADE assessment is shown in Table 4.

**TABLE 2:** Quality assessment of cohort studies and case–control studies with the Newcastle-Ottawa Scale (NOS).

References	Study design	Selection				Comparability		Exposure/outcome			Summary score
Chuang et al. (18)	Cohort	✩	✩	✩	✩	✩	✩	✩	✩	✩	9/9
Albrecht et al. (27)	Cohort	✩		✩	✩				✩	✩	5/9
Lu et al. (32)	Cohort	✩	✩	✩	✩	✩	✩	✩	✩	✩	9/9
Ozcaka et al. (33)	Case control	✩	✩		✩	✩	✩	✩	✩	✩	8/9
Crincoli et al. (34)	Case control	✩				✩	✩		✩	✩	5/9
Ergun et al. (44)	Case control	✩	✩			✩	✩	✩	✩	✩	7/9
Pedersen et al. (31)	Case control	✩				✩		✩	✩	✩	5/9

**TABLE 3:** Quality assessment of cross-sectional studies with the Agency for Healthcare Research and Quality (AHRQ) methodology checklist.

References	Study design	1	2	3	4	5	6	7	8	9	10	11	Summary score
Antoniazzi et al. (17)	Cross-sectional	1	1	1	0	1	1	1	1	0	1	0	8/11
Leung et al. (38)	Cross-sectional	1	1	0	0	0	1	1	1	0	0	0	5/11
Najera et al. (39)	Cross-sectional	1	1	0	0	0	1	1	0	0	0	0	4/11
Tervahartiala et al. (43)	Cross-sectional	1	1	0	0	0	0	0	0	0	0	0	2/11
Ambrosio et al. (29)	Cross-sectional	1	1	1	0	1	1	1	1	0	1	0	8/11
Marton et al. (35)	Cross-sectional	1	1	0	0	0	1	0	0	0	0	0	3/11
Kuru et al. (16)	Cross-sectional	1	1	0	0	0	1	1	1	0	0	0	5/11
Pedersen et al. (40)	Cross-sectional	1	1	1	0	0	0	1	0	0	0	0	4/11
Pedersen et al. (41)	Cross-sectional	1	1	1	0	1	1	1	0	0	0	0	6/11
Tseng (30)	Cross-sectional	1	1	0	0	0	0	1	0	0	0	0	3/11
Zoppo et al. (42)	Cross-sectional	1	1	0	0	0	1	0	0	0	0	0	3/11
Seck-Diallo et al. (36)	Cross-sectional	1	1	0	0	0	1	1	0	0	0	0	4/11
Pers et al. (37)	Cross-sectional	1	1	0	0	0	0	1	0	0	0	0	3/11
Rhodus and Michalowicz (28)	Cross-sectional	1	1	0	0	0	1	1	1	0	0	0	5/11

**TABLE 4:** The Grading of Recommendations Assessment, Development, and Evaluation (GRADE) assessment for the incidence of periodontal disease on Sjogren’s syndrome subjects.

Outcome	No. of studies	Study design	Certainty assessment					OR (WMD) [95% CI]	Certainty
			Risk of bias	Inconsistency	Indirectness	Imprecision	Other considerations		
Incidence of periodontal disease	5	Observational study	Not serious	Serious	Not serious	Not serious	None	OR = 2.12, 95% CI = 1.43–3.17	⊕⊕ LOW
BOP	7	Observational study	Not serious	Serious	Not serious	Not serious	None	WMD = 9.62, 95% CI = 3.43–15.80	⊕ VERY LOW
PI	12	Observational study	Not serious	Serious	Not serious	Serious	None	WMD = 0.15, 95% CI = −0.00–0.30	⊕ VERY LOW
GI	10	Observational study	Not serious	Serious	Not serious	Not serious	None	WMD = 0.28, 95% CI = 0.04–0.51	⊕ VERY LOW
PPD	12	Observational study	Not serious	Serious	Not serious	Serious	None	WMD = 0.22, 95% CI = −0.19–0.63	⊕ VERY LOW
CAL	6	Observational study	Not serious	Serious	Not serious	Serious	None	WMD = 0.40, 95% CI = −0.00–0.81	⊕ VERY LOW

OR, odds ratio; WMD, weighted mean difference.

### 3.2. Study of periodontal diseases in SS subjects

**FIGURE 2:**
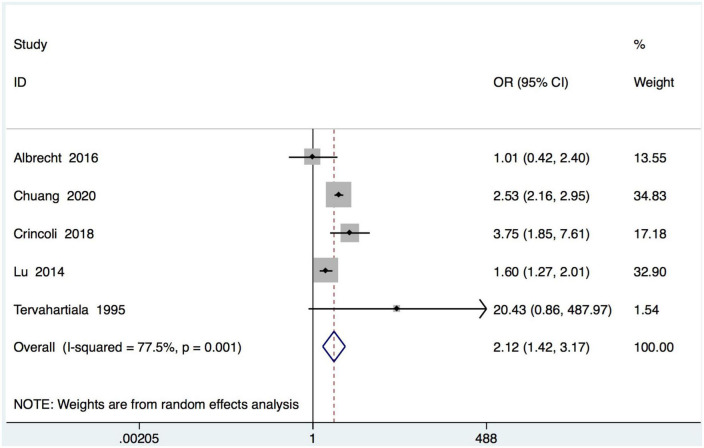
Forest plot for periodontal diseases in subjects with Sjogren’s syndrome (SS) and controls. OR, odds ratio; CI, confidence interval.

Five studies recording the incidence of periodontitis in SS subjects (Table 1). In the meta-analysis of these 5 studies, a positive association between SS and periodontal diseases was found and SS patients were more likely to be diagnosed with periodontal diseases than healthy controls (OR = 2.12, 95% CI = 1.43–3.17; 5 studies, 6927 participants; low certainty of evidence, I^2^ = 77.5%) (Figure 2).

**FIGURE 3:**
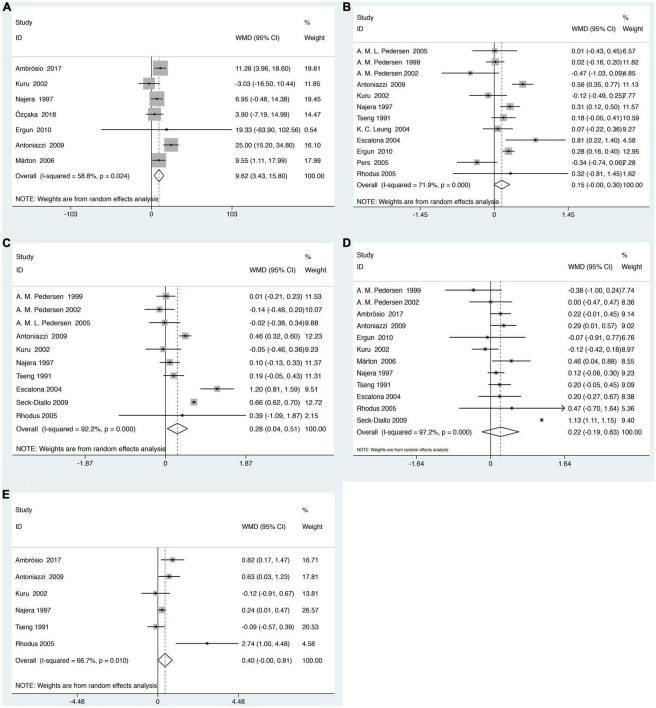
Forest plot for SS and clinical periodontal parameters scores **(A)**. Clinical periodontal parameters scores bleeding on probing (BOP) **(B)**. Clinical periodontal parameters scores plaque index (PI) **(C)**. Clinical periodontal parameters scores gingival index (GI) **(D)**. Clinical periodontal parameters scores probing pocket depth (PPD) **(E)**. Clinical periodontal parameters scores clinical attachment level (CAL). WMD, weighted mean difference; CI, confidence interval.

There are 16 studies reporting clinical periodontal parameters scores, which recorded in 455 subjects with SS and 397 healthy subjects (Table 1). Subjects with SS had significant difference in the clinical periodontal parameters scores BOP (WMD = 9.62, 95% CI = 3.43–15.80, I^2^ = 58.8%) and GI (WMD = 0.28, 95% CI = 0.04–0.51, I^2^ = 92.2%) was found. However, subjects with SS had no significant difference in the clinical periodontal parameters scores PPD (WMD = 0.22, 95% CI = −0.19–0.63, I^2^ = 97.2%). Finally, the relationship between the SS and the clinical parameters scores CAL (WMD = 0.40, 95% CI = −0.00–0.81, I^2^ = 66.7%) and PI (WMD = 0.15, 95% CI = −0.00–0.30, I^2^ = 71.9%) is not very clear (Figure 3).

### 3.3. Sensitivity analysis

**FIGURE 4:**
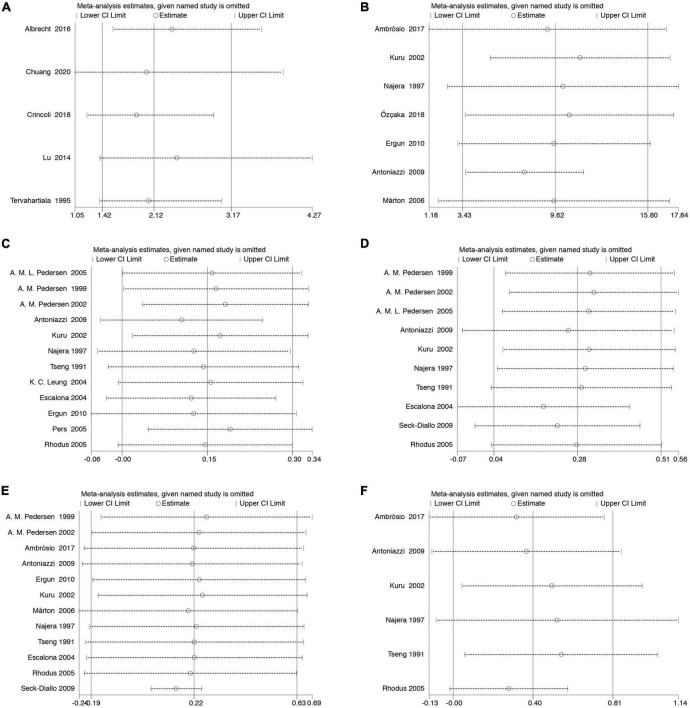
Sensitivity analysis. The pooled ORs (WMDs) and 95% CIs were stable after the deletion of each study in the analysis of the association between SS and controls **(A)**. The association between periodontal diseases and SS **(B)**. Clinical periodontal parameters scores BOP **(C)**. Clinical periodontal parameters scores PI **(D)**. Clinical periodontal parameters scores GI **(E)**. Clinical periodontal parameters scores PPD **(F)**. Clinical periodontal parameters scores CAL. CI, confidence interval.

Sensitivity analyses were performed by comparing the pooled sensitivity and specificity results when including and excluding studies with high risk of bias. There are no substantial changes in the meta-analysis results of the pooled ORs with corresponding 95% CIs (for the association between periodontal diseases and SS) and the pooled WMD s with corresponding 95% CIs (for the mean difference in the clinical periodontal parameter scores between subjects with SS and healthy controls), indicating that our meta-analysis is relatively stable (Figure 4).

### 3.4. Publication bias

**FIGURE 5:**
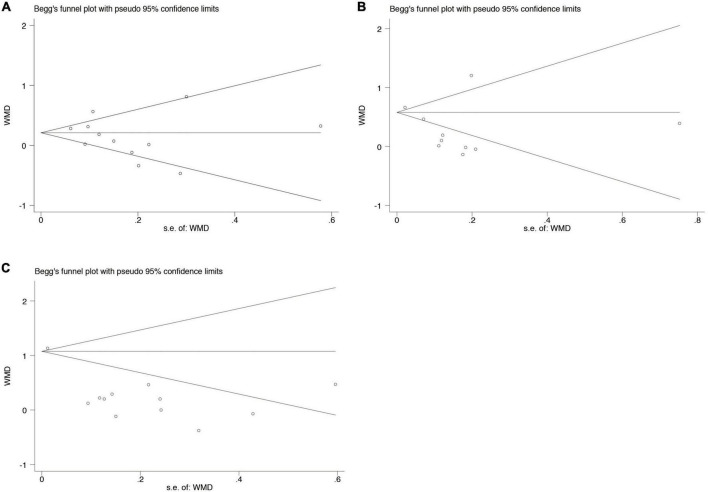
Begg’s funnel plot analysis to detect publication bias for the association between periodontal diseases and SS **(A)**. Clinical periodontal parameters scores PI **(B)**. Clinical periodontal parameters scores GI **(C)**. Clinical periodontal parameters scores PPD. WMD, weighted mean difference.

Potential publication bias was analyzed using Begg’s test in at least 10 studies included in the analyses. No significant publication bias was found in the WMD for clinical periodontal parameters scores PI (*p* = 0.537), GI (*p* = 0.721), and PPD (*p* = 0.086) between subjects with SS and healthy controls (Figure 5).

### 3.5. Meta-regression

Meta-regression was used to investigate potential sources of heterogeneity between the studies. We found that when the outcome variable was the percentage of periodontitis (*p* = 0.031), PI (*p* = 0.050), and GI (*p* < 0.001) age was one source of heterogeneity. When the outcome variable was PPD, the diagnostic criteria for SS was at least a source of heterogeneity (*p* = 0.025).

### 3.6. Trial sequential analysis

In meta-analyses, it is important to minimize the risk of false positive or false negative results. We therefore performed a TSA to control the risks for type I and type II errors and help to clarify whether additional trials are needed.

The results showed that for dichotomous variables, the meta-analysis can be declared as conclusive with regard to the anticipated effect leading to the required information size. That is, we can conclude that SS patients were more likely to be diagnosed with periodontal disease than healthy controls. Similarly, the continuous variable BOP also drew significant conclusions with enough information size (45).

**FIGURE 6:**
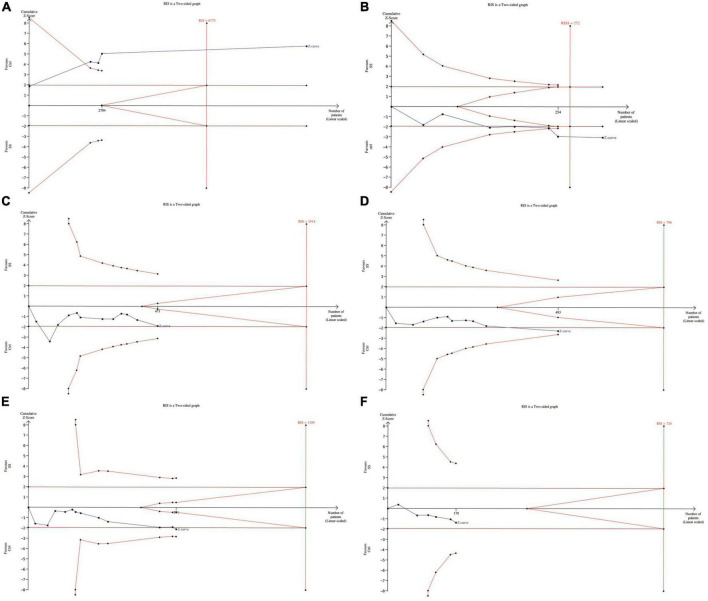
Trial sequential analysis of trials in subjects with SS **(A)**. The association between periodontal disease and SS **(B)**. Clinical periodontal parameters scores BOP **(C)**. Clinical periodontal parameters scores PI **(D)**. Clinical periodontal parameters scores GI **(E)**. Clinical periodontal parameters scores PPD **(F)**. Clinical periodontal parameters scores CAL. RIS, required information size.

For GI, although the meta-analysis came to a positive conclusion, it may have been declared not significant by TSA at this information size, meaning a false positive result. As for PI, PPD and CAL, irrelevant conclusions from meta-analysis are also inconclusive due to insufficient information. In fact, more tests need to be included for confirmation (46) (Figure 6).

## 4. Discussion

### 4.1. General interpretation of main results

In this meta-analysis of the studies on periodontal diseases and SS, we included 21 articles with 11435 participants meeting the inclusion criteria. GRADE Quality of evidence Indicates that the quality of evidence included in the study is not high. A positive association between periodontal diseases and SS was found. Subjects with SS had significantly higher clinical periodontal parameter scores BOP (WMD = 9.62, 95% CI = 3.43–15.80) and GI (WMD = 0.28, 95% CI = 0.04–0.51). However, there is no significant difference in the clinical periodontal parameters scores PI (WMD = 0.15, 95% CI = −0.00–0.30) and PPD (WMD = 0.22, 95% CI = −0.19–0.63) in SS patients. TSA trail showed that for dichotomous variables, the meta-analysis can be declared as conclusive with regard to the anticipated effect leading to the required information size. Similarly, the continuous variable BOP also drew significant conclusions with enough information size. For GI, PI, PPD, and CAL, more tests need to be included for confirmation.

### 4.2. Strengths and limitations of the review

There are several strengths in Our meta-analysis. First, we searched all relevant databases as thoroughly as possible to include more studies that met the criteria. Second, we performed GRADE evidence quality grading to reflect the accuracy of the effect estimates. Third, we conducted TSA Trail to manage the risk of type I and II errors and to help determine whether additional trials are needed. Therefore, our analysis could well reflect the trial effect and illustrate the clinical guiding significance of the indicator.

There are also limitations in our study. First, the results had relatively high statistical heterogeneity (I^2^ = 77.5%, 58.8%, 71.9%, 92.2%, 97.2%, and 66.7%) across the studies. Although we tried meta-regression to explore the sources of heterogeneity and obtained some sources of heterogeneity, we did not find more sources of heterogeneity due to the lack of more detailed description of research characteristics in the included studies. Second, although some positive conclusions are reached in TSA Trail results, some conclusions are still unstable and lack sufficient information. Therefore, the current conclusions may change with the increase of studies. Third, because a small part of the included study data came from dental clinics, patients from dental clinics may have some dental diseases or have some symptoms, so these patients are more likely to suffer from periodontal disease. In contrast, patients from other places such as Sjogren’s Syndrome Service of the Hospital Clinics showed no such bias. All in all, the results we get may lean toward “correlation.” In addition, we tried to search the studies on the incidence of Sjogren’s syndrome in patients with periodontal disease. Unfortunately, due to the retrieval of only two articles (47, 48), meta-analysis could not be conducted, and further relationship could not be obtained. However, both papers concluded that patients with periodontal disease were more likely to have Sjogren’s syndrome. To some extent, we believe that such conclusions can help to prove the relationship between Sjogren’s syndrome and periodontal disease. Our results should be interpreted with caution considering the existence of multiple conditions.

### 4.3. Implications for practice and future research

According to previous studies, periodontal diseases and SS have been considered to be related (47), which has been shown by quite a few studies. SS patients had a higher incidence of tooth loss, a higher risk ratio of undergoing one or more tooth extractions, and significantly more SS patients were edentulous, which may be related to reduced salivary secretion (49). SS can lead to a decrease in saliva flow and a change in its composition, which affects the removal of bacteria in the oral cavity and thus facilitates the accumulation of plaque on the surface of teeth, which contributes to the deterioration of clinical periodontal parameters and, in severe cases, periodontal diseases (15). It has also been reported that the salivary glands of patients with SS are characterized by lymphocytes gathering around the salivary ducts, which plays an important role in driving the local immune response (50). The capillaries of gingival microcirculation in patients with SS have been changed, which may be related to the occurrence of periodontal diseases (51). Moreover, it has been found that the patients with Sjogren’s syndrome had higher serum concentrations of anti-periodontal pathogens such as *Aggregatibacter actinomycetemcomitans* and *Porphyromonas gingivalis*. Furthermore, alterations in cytokine networks may act as an intermediate factor between periodontal diseases and SS. More inflammatory cytokines were also found in the oral cavity of patients with SS, which can stimulate the differentiation of immune cells, thus affecting the occurrence and development of periodontal diseases (17, 52). However, the underlying pathophysiological mechanism between periodontal diseases and Sjogren’s syndrome are so complex that by now, it has not been fully understood (47). Unfortunately, we still have no conclusive evidence to judge the relationship between periodontal disease and Sjogren’s syndrome.

Despite the lack of clear evidence, our meta-analysis still has its value for the clinical prevention and treatment of periodontal diseases and SS. The first consultation department of many SS patients is the dental department because dry mouth and dental caries are the initial symptoms of them. This meta-analysis is the first to explore the correlation between periodontal diseases and SS from the perspective of dentists and patients. From the perspective of dentists, the periodontal condition of SS patients should also be paid attention to when they are diagnosed and treated. If timely measures are taken to improve the periodontal condition, SS patients are expected to reduce or even avoid the progressive loss of periodontal tissue. From the patient’s point of view, the symptoms of periodontal disease and SS may serve as reminders of each other, and patients should pay close attention to the other when one is diagnosed. We believe that the treatment of SS is helpful in alleviating periodontal diseases, and the awareness of patients and clinicians on SS may contribute to the successful treatment of periodontal diseases. Future scientific studies should further verify the correlation between these two diseases and explore the mechanism and molecular pathway of their association.

## 5. Conclusion

This systematic review indicated that SS patients were more likely to be diagnosed with periodontal diseases, and the periodontal index is more likely to be abnormal, suggesting that the treatment of SS has a positive effect on periodontitis.

## Data availability statement

The original contributions presented in this study are included in the article/Supplementary material, further inquiries can be directed to the corresponding author/s.

## Author contributions

BY and XP put forward the ideas, searched the literature, filtered the information, analyzed the data, and wrote the manuscript. JG prepared the figures and tables and checked for errors. XuL, XiL, YW, ZC, and BC reviewed the manuscript, put forward the constructive comments, and participated in its design and coordination. All authors contributed to the article and approved the submitted version.
