# Supplementary material for: The association of periodontal diseases and Sjogren’s syndrome: A systematic review and meta-analysis
Source: Front Med (Lausanne). 2023 Jan 5;9:904638. doi: 10.3389/fmed.2022.904638 (PMC9851607; doi:10.3389/fmed.2022.904638)
Supplement: Supplementary file 1 [file Table_1.DOCX]

**Supplementary Table 1** Search strategies for databases.

| Database | Date of coverage | Search strategy |
| --- | --- | --- |
| **Pubmed** | until November 24, 2021 | #1(Sjögren’s syndrome [MeSH Terms]) OR (Sjogrens syndrome [MeSH Terms]) OR (Sjogren’s syndrome [MeSH Terms]) OR (Sjogren syndrome [MeSH Terms]) OR (Sicca syndrome [MeSH Terms]) OR (SS [MeSH Terms]) OR (Sjögren’s syndrome [Title/Abstract]) OR (Sjogrens syndrome [Title/Abstract]) OR (Sjogren’s syndrome [Title/Abstract]) OR (Sjogren syndrome [Title/Abstract]) OR (Sicca syndrome [Title/Abstract]) OR (SS [Title/Abstract])  #2 (periodontal disease [MeSH Terms]) OR (periodontal diseases [MeSH Terms]) OR (periodontitis [MeSH Terms]) OR (gingivitis [MeSH Terms]) OR (periodontal disease [Title/Abstract]) OR (periodontal diseases [Title/Abstract]) OR (periodontitis [Title/Abstract]) OR (gingivitis [Title/Abstract])  #3 #1 AND #2 |
| **EMBASE** | until November 24, 2021 | #1 'sjogrens syndrome' OR 'sjogren syndrome'/exp OR 'sicca syndrome'/exp OR 'ss'  #2 sjogrens syndrome.mp. OR sjogren syndrome.mp. OR sicca syndrome.mp. OR ss.mp.  #3 'periodontal disease' OR 'periodontal diseases'/exp OR 'periodontitis'/exp OR 'gingivitis'/exp  #4 periodontal disease.mp. OR periodontal diseases.mp. OR periodontitis.mp. OR gingivitis.mp.  #5 #1 OR #2  #6 #3 OR #4  #7 #5 AND #6 |
| **Web of Science** | until November 24, 2021 | #1 TI=(Sjögren’s syndrome) OR TI=(Sjogrens syndrome) OR TI=(Sjogren’s syndrome) OR TI=(Sjogren syndrome) OR TI=(Sicca syndrome) OR TI=(SS)  #2 TS=(Sjögren’s syndrome) OR TS=(Sjogrens syndrome) OR TS=(Sjogren’s syndrome) OR TS=(Sjogren syndrome) OR TS=(Sicca syndrome) OR TS=(SS)  #3 TI=(periodontal disease) OR TI=(periodontal diseases) OR TI=(periodontitis) OR TI=(gingivitis)  #4 TS=(periodontal disease) OR TS=(periodontal diseases) OR TS=(periodontitis) OR TS=(gingivitis)  #5 #1 OR #2  #6 #3 OR #4  #7 #5 AND #6 |
| **Cochrane Library** | until November 24, 2021 | #1 (Sjögren’s syndrome):ti,ab,kw OR (Sjogrens syndrome):ti,ab,kw OR (Sjogren’s syndrome):ti,ab,kw OR (Sjogren syndrome):ti,ab,kw OR (Sicca syndrome):ti,ab,kw OR (SS):ti,ab,kw  #2 (periodontal disease):ti,ab,kw OR (periodontal diseases):ti,ab,kw OR (periodontitis):ti,ab,kw OR (gingivitis):ti,ab,kw  #3 #1 and #2 |
